# Screening of breast cancer in higher-risk Taiwanese women using contrast-enhanced mammography

**DOI:** 10.1016/j.heliyon.2025.e41851

**Published:** 2025-01-10

**Authors:** Chen-Pin Chou, Yu-Ting Hong, Yun Lin, Pei-Ying Lin

**Affiliations:** a Radiology Department, Kaohsiung Veterans General Hospital, Kaohsiung, Taiwan, ROC; b Department of Medical Laboratory Science and Biotechnology, Fooyin University, Kaohsiung, Taiwan, ROC; c Department of Pharmacy, College of Pharmacy, Tajen University, Pingtung, Taiwan, ROC

**Keywords:** Breast cancer, Mammography, Contrast-enhanced mammography, Higher-risk, BI-RADS category

## Abstract

**Background:**

To assess the diagnostic performance of contrast-enhanced mammography (CEM) in screening higher-risk Taiwanese women for breast cancer.

**Methods:**

We conducted a prospective study at a Taiwanese medical center from 2019 to 2021. The study compared imaging techniques for breast cancer screening in women with a personal history of precancer or cancer, or a family history (first-degree relatives). The study included breast ultrasound (US) and the CEM combo, which consists of digital mammography (DM), digital breast tomosynthesis (DBT), and CEM. Diagnostic accuracy was compared using the receiver operating characteristic (ROC) curve.

**Results:**

The study included 308 women, average age 52.1, with 86 % having familial breast cancer history and 14 % personal breast cancer or pre-cancerous histories. Approximately 19.5 % had lesions rated BI-RADS 4 or 5. Biopsies were performed on 56 women based on lesions detected by the CEM combo. Additionally, 60 biopsies were due to abnormalities found via DM or DBT (n = 20) or US (n = 40). Breast cancer was confirmed in 8 women post-biopsy. The CEM combo showed a sensitivity of 87.5 %, significantly higher than DM (50 %), DBT (50 %), and US (25 %). The ROC curve area for CEM was 0.85, outperforming DM (0.65), DBT (0.63), and US (0.55), with all comparisons statistically significant (p < 0.05). Lesions detected solely by DM, DBT, or US did not yield any cancer cases.

**Conclusions:**

CEM effectively detects breast cancer in higher-risk Taiwanese women, but further research is needed to refine biopsy recommendations.

## 1 Introduction

Mammography is the primary imaging method used for breast cancer screening, and it is the only imaging test that has been shown to decrease breast cancer mortality [1,2]. Despite its effectiveness of breast cancer screening in Western and Asian countries, mammography has a drawback in its sensitivity, particularly in women with dense breasts. Studies have shown that mammography's sensitivity in women with extremely dense breast tissue can be as low as 30 % [3].

Breast MRI is a sensitive imaging technique that can be used to detect breast malignancies [4], but it is typically recommended only for screening higher-risk women, such as those with a personal history of breast cancer, dense breasts, or those diagnosed before the age of 50, due to its high cost and limited availability [[5], [6], [7], [8]]. Contrast-enhanced mammography (CEM) is a novel advancement in mammography techniques that utilizes contrast agent to demonstrate the vascularity of breast cancers. A preliminary study found that CEM was accurate in detecting two invasive cancer, highlighting its potential as a diagnostic tool compared to breast MRI, which identified all three malignancies [9]. In a subsequent study involving women at increased risk, 904 patients underwent baseline CEM, which demonstrated a sensitivity of 50.0 % for digital mammography (DM) and 87.5 % for CEM [10].

The application of CEM for screening higher-risk Taiwanese women has not been studied. This study investigates the application of CEM for breast cancer screening in higher-risk Taiwanese women, comparing its effectiveness to other mammography techniques and ultrasound (US) imaging tests.

## 2 Methods

### 2.1 Study participants

A prospective study, approved by the Institutional Review Board of Kaohsiung Veterans General Hospital (IRB: VGHKS18-CT11-17, Clinical Trials: NCT05797129), was conducted from January 2019 to December 2021. All participants provided written informed consent prior to inclusion, adhering to the ethical standards outlined in the Declaration of Helsinki. During this period, 351 women participated in screenings using CEM. Inclusion criteria comprised women aged 30 years or older who had undergone a partial mastectomy for invasive breast cancer or ductal carcinoma in situ (DCIS), or those with a family history of breast cancer involving one or more first-degree relatives. Participants completed a questionnaire on breast cancer risk factors, confirmed the absence of contrast material allergies, had normal renal function, and consented to contrast agent administration. Exclusion criteria included women younger than 30 years, individuals undergoing CEM for diagnostic purposes (rather than screening), and patients with negative CEM results without at least 12 months of follow-up. Forty-three participants were excluded due to incomplete imaging assessments or insufficient follow-up data, leaving 308 CEM examinations in the final analysis.

### 2.2 Risk score calculation

The Breast Cancer Risk Assessment Tool (BCRAT), available on the US National Cancer Institute's website, was used to calculate the risk of breast cancer. The BCRAT, based on the Gail model, allows for a comprehensive evaluation of specific risk variables relevant to the possibility of breast cancer. The database provides the potential for risk assessment within the Chinese subgroup demography. However, this feature is not essential. The BCRAT estimates a person's lifetime risk of breast cancer using factors such as age, race/ethnicity, personal and family medical history, and other known risk factors. The risk assessment is conducted using an established questionnaire. Individuals diagnosed with invasive breast cancer or ductal carcinoma in situ (DCIS) were excluded from the risk score calculation as the Gail model, used to stratify high-risk populations, is not intended for risk assessment in this group.

### 2.3 CEM parameters

**Fig. 1:**
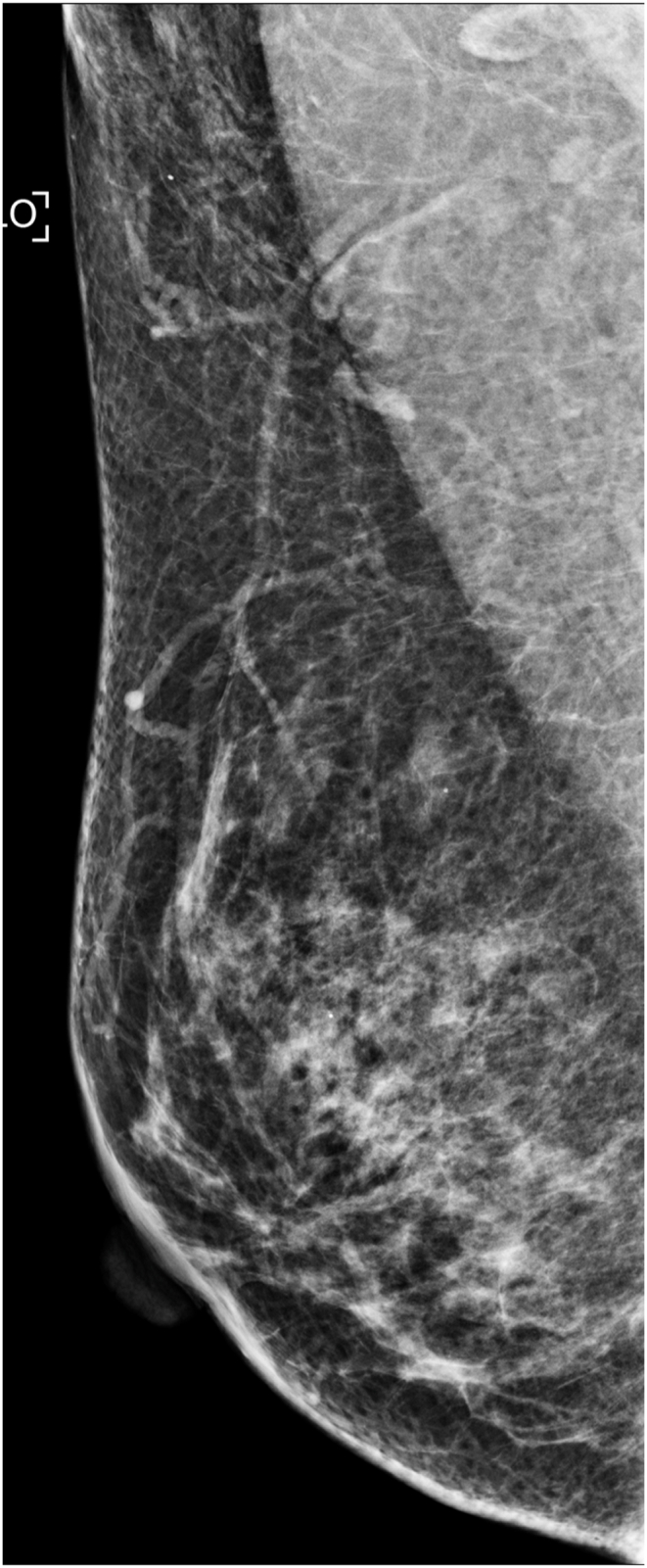

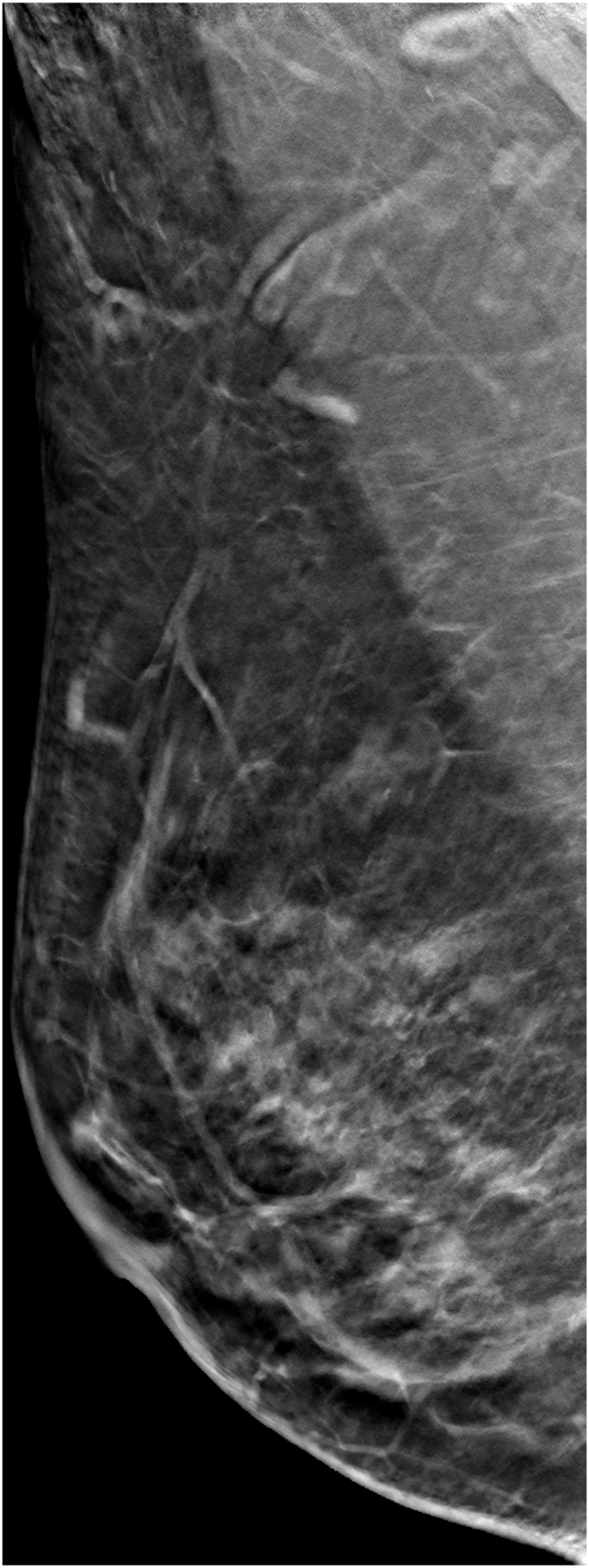

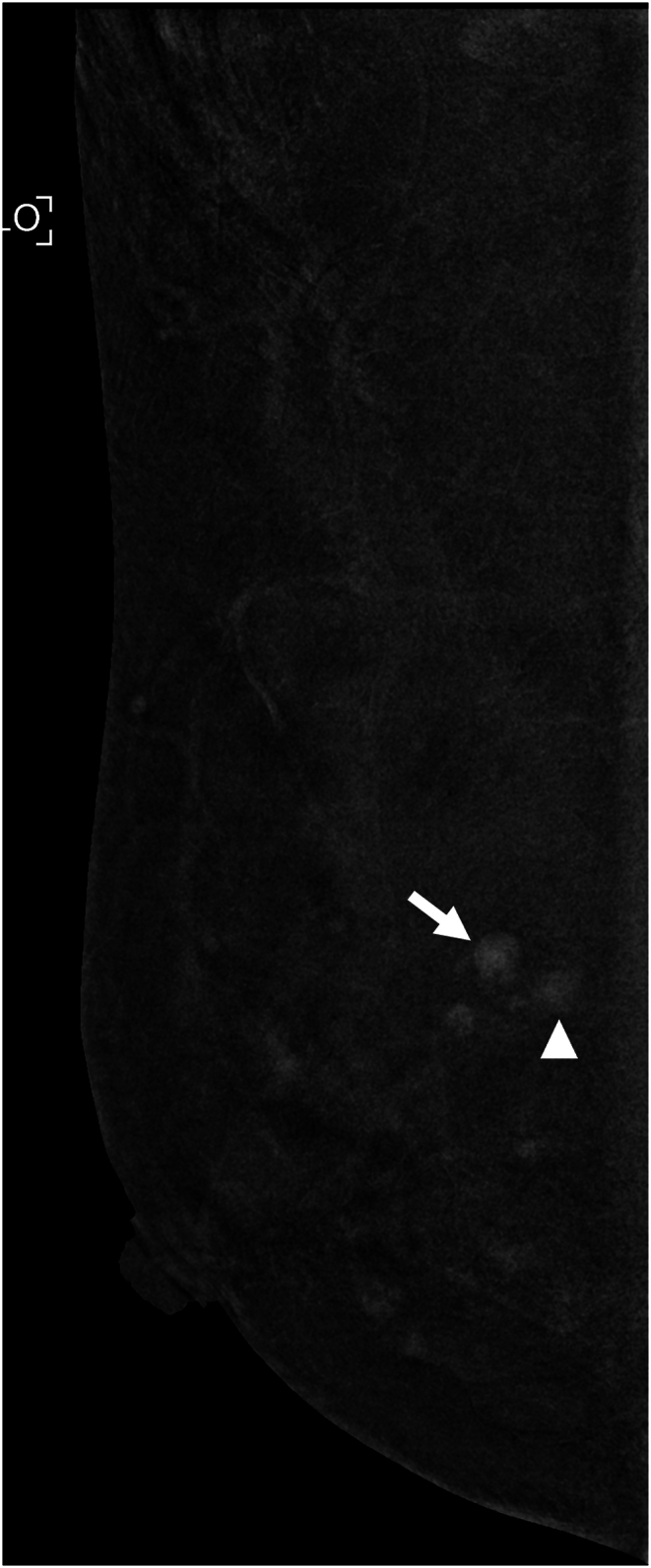

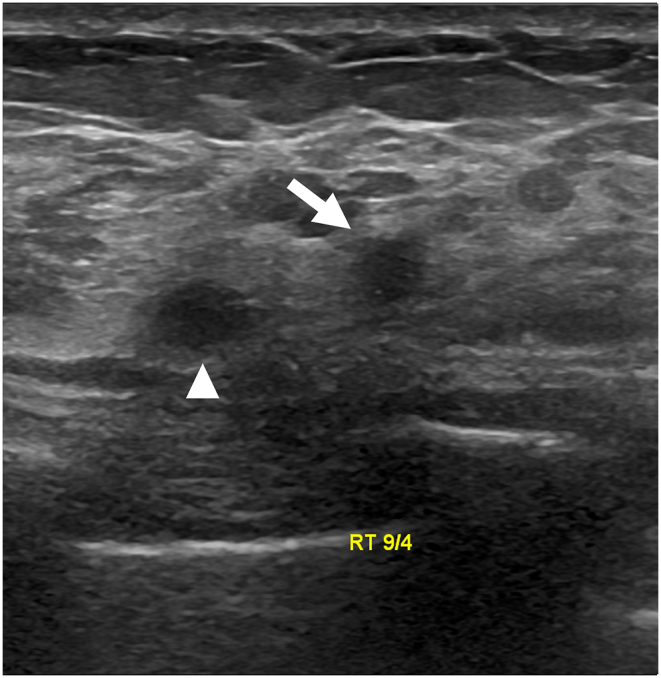
A 57-year-old woman with a history of atypical ductal hyperplasia (ADH) underwent breast imaging screening. DM and DBT were reported as negative findings for malignancy, and screening US reported two probably benign nodules in right breast 9 o'clock direction and 4 cm from nipple. However, CEM identified two suspicious enhancing lesions in the right breast. One was a 0.8 cm invasive breast cancer (arrow), and the other was a benign breast adenosis (arrowhead). These two lesions were confirmed by separate US-guided core-needle biopsies. (a) DM, (b) DBT, (c) CEM, and (d) Screening US.

**Fig. 2:**
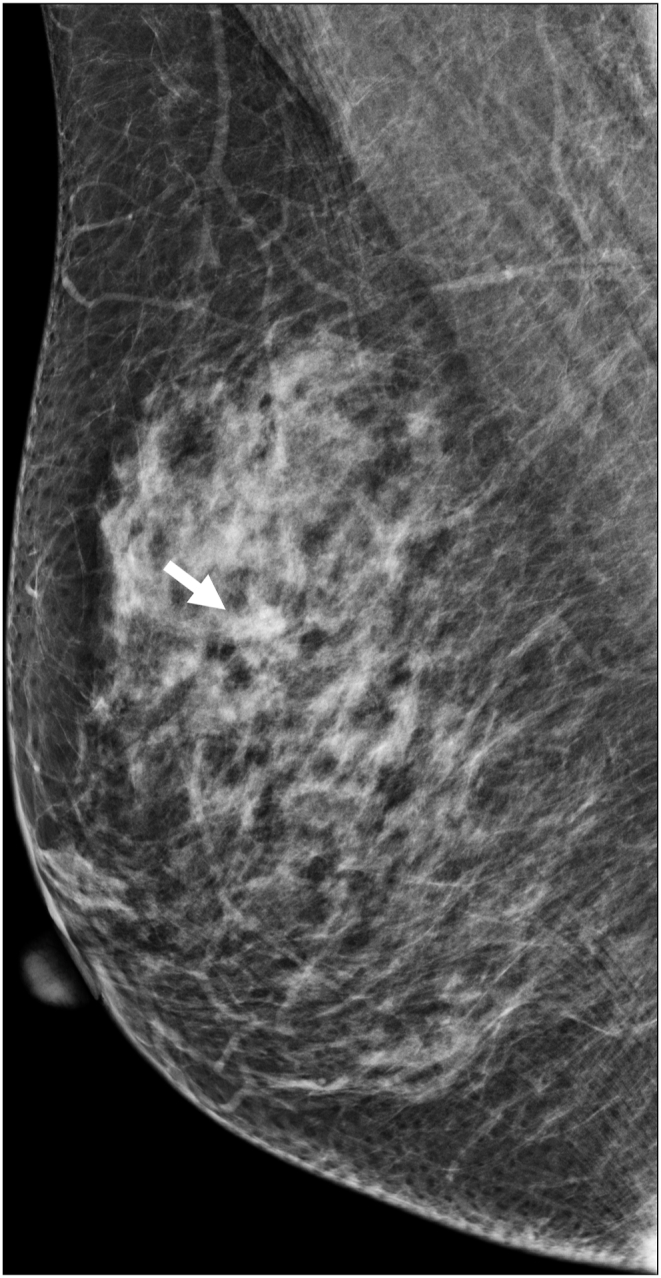

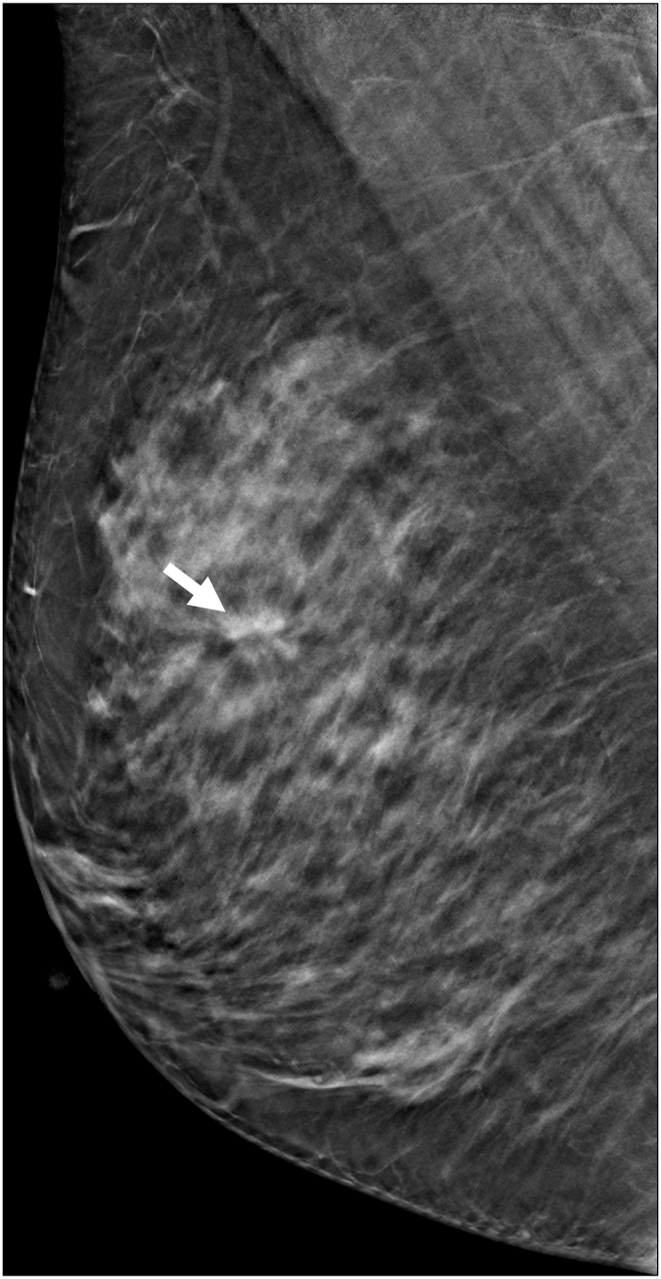

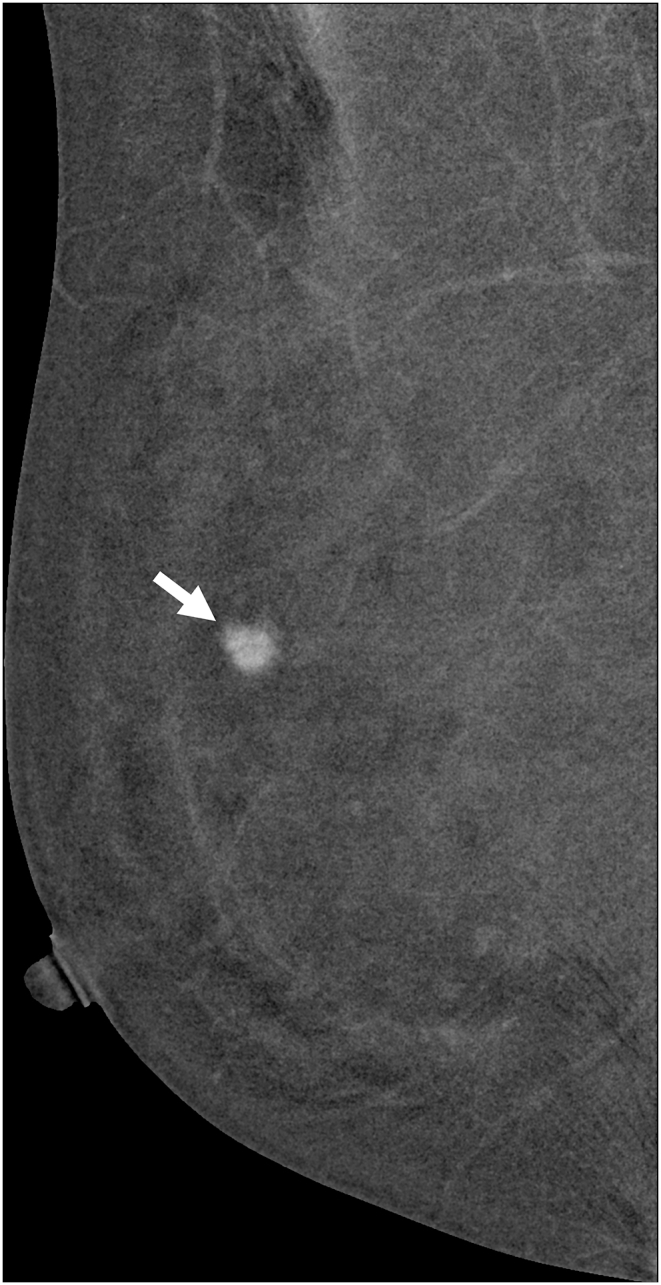

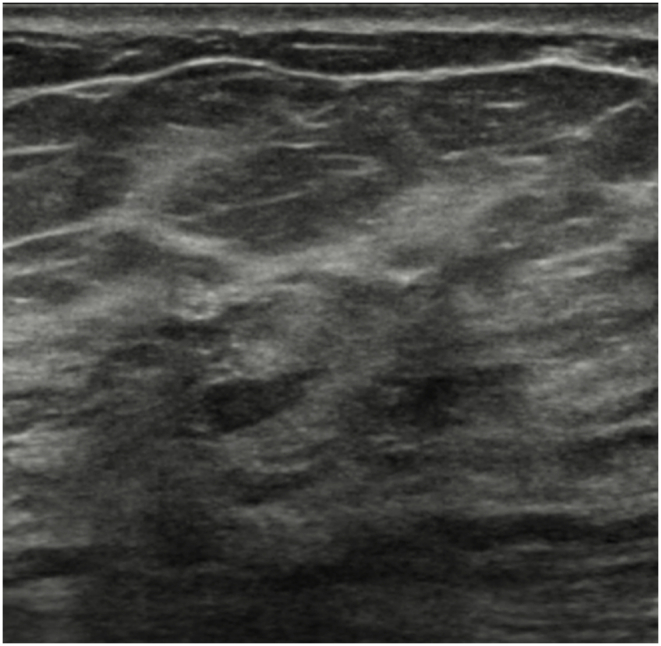

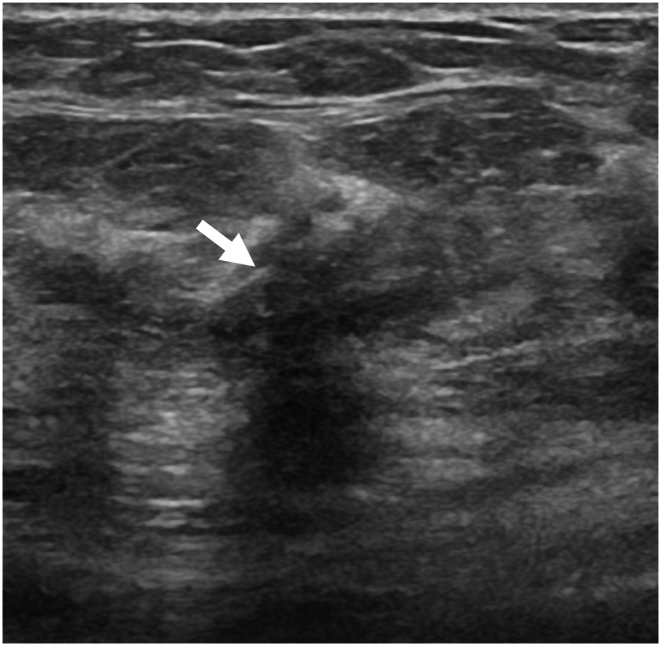
A 51-year-old woman with a family history of breast cancer in her mother received breast imaging screening. All imaging techniques including DM, DBT, CEM, and second-look US prior to core-needle biopsy revealed a 0.9 cm invasive breast cancer (arrow). However, the initial screening breast US showed no suspicious findings. (a) DM, (b) DBT, (c) CEM, (d) Screening US, and (e) Second-look US.

The technologists performed CEM exams using a dual-energy breast mammography system (Selenia Dimensions; Hologic, Bedford, USA). An automated injector with an 18-gauge peripheral intravenous needle was used to administer a 3 mL/s intravenous injection of 1.5 mL/kg of iodine contrast agent (Omnipaque 350; GE Healthcare) with a maximum dose of 100 mL. The breast was not compressed during the injection. Approximately 2 min after injection, each woman was positioned similarly to a standard mammography exam, and imaging was started 2 min after injection. Each projection was imaged with two exposures, low energy exposure (Rh or Ag filtration; 26–32 kVp) and high energy exposure (45–49 kVp). Using I-view post-processing software (Hologic), subtraction images were obtained from the low and high energy acquisitions (Fig. 1, Fig. 2(A - E)), which emphasized the accumulation of the injected iodine contrast agent in the lesion. The generated images included DM, DBT, and CEM in the craniocaudal (CC) view and mediolateral oblique (MLO) view. The low-energy CEM image was considered as the DM image. The average radiation dose per imaging session for each breast tomosynthesis with CEM, with a breast compression thickness of 4.2 cm, was found to be less than 3.0 mGy [11].

### 2.4 Breast ultrasound

The US screenings of both breasts were performed on the same day as the CEM exams using GE E9 machines (General Electric Medical Systems, Milwaukee, WI, USA). Sonographers, with 5–25 years of specialized expertise in breast US, performed these screening exams without knowing the mammography findings. Sonographers used radial and anti-radial scanning techniques to visualize intraductal lesions. All mass lesions were imaged in two orthogonal planes and marked on the technologist's diagram. The axilla was routinely scanned, and any lymph nodes found were documented. Images of all four quadrants and the subareolar region should be routinely obtained. The images were stored and transferred to a radiologist for assessment.

### 2.5 Suspicious lesion evaluation

Two radiologists, each with 18 and 22 years of experience, respectively, in screening and diagnostic DM and 7 years in CEM, independently interpreted DM, DBT and CEM, before collaboratively reviewing these interpretations on a dual-monitor workstation (SecurViewDx, Hologic MA). Consensus was defined as both radiologists agreeing on the interpretation; in cases of initial disagreement, a joint review session was held to discuss and resolve the differences until agreement was reached. Images were classified using the American College of Radiology's Breast Imaging Reporting and Data System (ACR BI-RADS), 5th Edition [12]. The images were classified by breast density (A through D) and diagnostic category (1 through 5). Extremely dense breasts' (category D) can obscure lesion detection on DM, thereby impacting diagnostic accuracy. The images are analyzed for background parenchymal enhancement (BPE), categorized into four levels (minimal, mild, moderate, and marked), which influences lesion visibility. In this research, the BI-RADS 0 category, indicating an undetermined risk of cancer, was not applicable due to statistical requirements.

Radiologists focused on identifying abnormalities by evaluating their shape, margins, and enhancement patterns. A crucial part of interpretation includes comparing the current CEM images with previous mammograms to track changes and identify persistent or new lesions. The review of CEM combo adhered to a predefined sequence, starting with DM, followed by DBT, and CEM, incorporating information from previous imaging to refine suspicion levels and potential additional sensitivities of each modality. While assessing DBT and CEM, the corresponding DM was accessible. During the CEM combo analysis, both DM and DBT were present for a direct side-by-side comparison.

The breast US diagnosis was determined by one of seven radiologists, each with 3–22 years of experience in breast US, who independently interpreted the US images without knowledge of the CEM combo findings. The readers characterized lesions detected in DM and DBT by features such as calcified or non-calcified lesions. Precise measurements were taken of all identified lesions, serving as crucial information for evaluating their severity and stage. With stereotactic biopsy not available at our institution, suspicious CEM findings without corresponding US features were evaluated by breast MRI for a final risk assessment.

### 2.6 Contrast agent allergic reactions

Any women who had a reaction to the contrast agent were evaluated by a radiologist. The reaction to the contrast agent was recorded and classified as mild, moderate, or severe according to guidelines outlined in the American College of Radiology Imaging Agent Manual [13].

### 2.7 Statistical analysis

We computed diagnostic metrics, including sensitivity, specificity, positive predictive value (PPV), and negative predictive value (NPV). BI-RADS categories 1–3 were classified as negative and categories 4 and 5 as positive results. Differences in diagnostic parameters between the diagnostic modalities were assessed using McNemar's test, which provides a statistical comparison of the diagnostic values. The receiver operating characteristic curves (ROC) of the imaging modalities were calculated using the BI-RADS categories ordinal scale (categories 1–5). The corresponding area under curve (AUC) values were also calculated. The statistical analysis was performed using MedCalc software (version 16.8, MedCalc Software). A p-value <0.05 was considered statistically significant.

## 3 Results

### 3.1 Patient characteristics

The final study cohort consisted of 308 higher-risk women (mean age 52.1 years, range: 35–71) who underwent CEM combo and bilateral breast US exams (Table 1). Most patients were older than 50 (56.5 %), with dense breasts (83.8 %) and a family history of breast cancer (86.0 %). All patients under 40 year-old came for screening primarily due to a family history. Only a minority (14.0 %) presented personal risk factors for breast cancer due to previous surgery. According to the Gail model, the women in this study who weren't previously diagnosed with breast cancer had an average lifetime risk of 13.4 ± 5.2. One woman in a group of 308 patients (0.3 %) experienced a mild adverse reaction to a contrast agent, manifested as a localized skin rash that resolved within a few days following treatment with oral chlorpheniramine maleate.

**Table 1:** Clinical characteristics of the women included in the study (n = 308).

Characteristics	Number (%)
Age distribution (years)	
Age≦50	134 (43.5)
Age >50	174 (56.5)
Breast density (5th ACR BI-RADS)	
Non-dense (BI-RADS A or B)	50 (16.2)
Dense (BI-RADS C or D)	258 (83.8)
Risk factors of breast cancer	
Personal risk	43 (14.0)
Family risk	265 (86.0)
BI-RADS category (based on CEM combo)	
1	160 (51.9)
2	42 (13.6)
3	47 (15.3)
4	56 (18.2)
5	3 (1.0)

Note: Data in parentheses are percentages. BI-RADS=Breast Imaging Reporting and Data System, ACR= American College of Radiology, CEM=Contrast-enhanced mammography.

### 3.2 BI-RADS assessment

The distribution of BPE patterns of CEM in our cohort was as follows: minimal in 24 % of patients, mild in 39 %, moderate in 29 %, and marked in 8 %. The BI-RADS assessment of the CEM combo resulted in the following: 202 women (51.9 %) had BI-RADS 1 or 2, 46 patients (14.9 %) had BI-RADS 3, and 59 patients (19.5 %) had BI-RADS 4 or 5. The suspicious lesions (BI-RADS 4 and 5) were identified in DM, DBT, and US at 21.1 %, 24.6 %, and 14.3 %, respectively. All 59 women with BI-RADS 4 or 5 lesions identified through the CEM combo underwent diagnostic imaging tests, including targeted breast ultrasound and diagnostic spot mammography, to assist radiologists in performing further imaging-guided biopsies. Six patients had dynamic contrast-enhanced magnetic resonance imaging (DCE-MRI) to look more closely at lesions that were found to be suspicious by CEM but not clearly seen by US. Following MRI exams, three patients were re-categorized as MRI BI-RADS 3 as probably benign, while biopsies were performed on the other three MRI BI-RADS 4 patients using an MRI-guided biopsy.

### 3.3 Pathology diagnosis

After the CEM screening and MRI workups, 56 (18.2 %) individuals with CEM BI-RADS 4 or 5 underwent a biopsy procedure, and 7 breast cancer cases were confirmed. Based on BI-RADS results from DM and DBT alone (n = 20), and US alone (n = 40), an additional 60 patients with CEM BI-RADS 1–3 were also recommended for biopsy, and no cancers were found in pathology diagnosis. Of the total 116 women who were recommended for biopsy, 40 had tomosynthesis-guided procedures to examine microcalcifications (n = 33) and architectural distortion (n = 7). Additionally, 73 women received a US-guided biopsy. Three women underwent MRI-guided biopsies due to lesions only visible on CEM and MRI. One woman with CEM BI-RADS 2 had breast cancer found due to a suspicious lesion on a self-referred breast MRI. The other 247 women with CEM BI-RADS 1–3 did not develop breast cancer within 12 months.

### 3.4 Post-screening surveillance strategy

Post-CEM surveillance for every 6-month and 12-month follow-up in CEM BI-RADS 3 and BI-RADS 1 or 2 patients, respectively. Of 248 women who were classified as CEM combo BI-RADS 1–3, one patient had a self-referred breast MRI and a BI-RADS 4 MRI suspicious lesion was found. No new breast cancer diagnoses were made during the clinical and imaging surveillance follow-up of the remaining 247 women.

### 3.5 Characteristics of breast cancer patients

Among 116 patients, 8 (6.9 %) were diagnosed with breast cancer, while 108, (93.1 %) had benign breast diseases. No cancers were found in a subgroup of 60 patients with BI-RADS 1–3 on CEM despite biopsy indications from other modalities (DM, DBT, US). Seven cancers were identified in the 56 patients with BI-RADS 4–5 on CEM. One CEM negative case had a self-referred breast MRI exam, which revealed a 0.7 cm cancer missed by CEM and later detected on MRI within 12 months. Overall, the study revealed that a total of 10 breast cancers were identified in 8 women, including 2 ductal carcinomas in situ (DCIS) and 8 invasive ductal carcinomas (IDC) (Table 2). Two cases of multifocal breast cancer were observed: one patient with bilateral IDCs and another with concurrent IDC and DCIS in the same breast. The sizes of the identified invasive breast cancers were found to range from 0.7 cm to 1.5 cm. There were three calcified lesions with microcalcifications and seven non-calcified lesions. Luminal A, a specific molecular subtype, was the most common, being present in 60 % of the cases. No axillary or distant metastases were identified at surgery, imaging, or clinical assessment. In a study of 8 breast cancers, the CEM combo identified 7 cases, exceeding the individual detection rates of DM (4 cases), DBT (4 cases), and US (2 cases).

**Table 2:** Comprehensive evaluation of 10 breast cancer lesions within 8 women from a screening study, including one diagnosis via self-referred breast MRI.

Case No.	Lesion No.	Age (year)	Risk Factors	Breast Density	Lesion type	Histology molecular subtype^b^	Invasive Cancer size (cm)	Tumor Grade^c^	Positive Modality
1	1	39	PH	ED	Cal	DCIS	NA	II	DM/DBT/CEM
						Luminal A			
2^a^	2	56	FH, PH	HD	Cal	DCIS	NA	III	DM/DBT/CEM
						Luminal A			
2^a^	3	56	FH, PH	HD	Non-cal	IDC	1.5	II	DM/DBT/CEM/US
						Luminal B			
3	4	57	PH	HD	Non-cal	IDC	0.8	I	CEM
						Luminal B			
4^a^	5	51	FH, PH	HD	Non-cal	IDC	1.2	III	CEM
						Luminal B			
4^a^	6	51	FH, PH	HD	Non-cal	IDC	1.0	III	CEM
						Luminal B			
5	7	49	FH	HD	Non-cal	IDC	1.4	I	DM/DBT/CEM/US
						Luminal A			
6	8	46	FH	HD	Non-cal	IDC	0.7	II	MRI
						Luminal A			
7	9	53	FH	HD	Non-cal	IDC	1.2	I	CEM
						Luminal A			
8	10	58	FH	SFG	Cal	IDC	0.9	II	DM/DBT/CEM
						Luminal A			

Note: The following abbreviations are used in this table: FH=Family history, PH=Personal history, ED = Extremely dense, SFG=Scattered fibroglandular density, HD=Heterogeneously dense, DCIS = Ductal carcinoma in situ, IDC=Invasive ductal carcinoma, NA=Not applicable due to non-invasive cancer, Cal = Calcified lesion, Non-cal = Non-calcified lesion, DM = Digital mammography, DBT = Digital breast tomosynthesis, and CEM=Contrast-enhanced mammography.

a Patients 2 and 4 have multiple lesions.

b Molecular subtype criteria consider ER and PR to be positive if more than 1 % of the stained nuclei are positive; the cut-off point for Ki-67 is 20 %, and HER2 expression is considered positive if amplified with fluorescent in situ hybridization.

c Tumor grades range from grade I being low, grade II being intermediate, to grade III being high.

### 3.6 Diagnostic performance of CEM

**Fig. 3:**
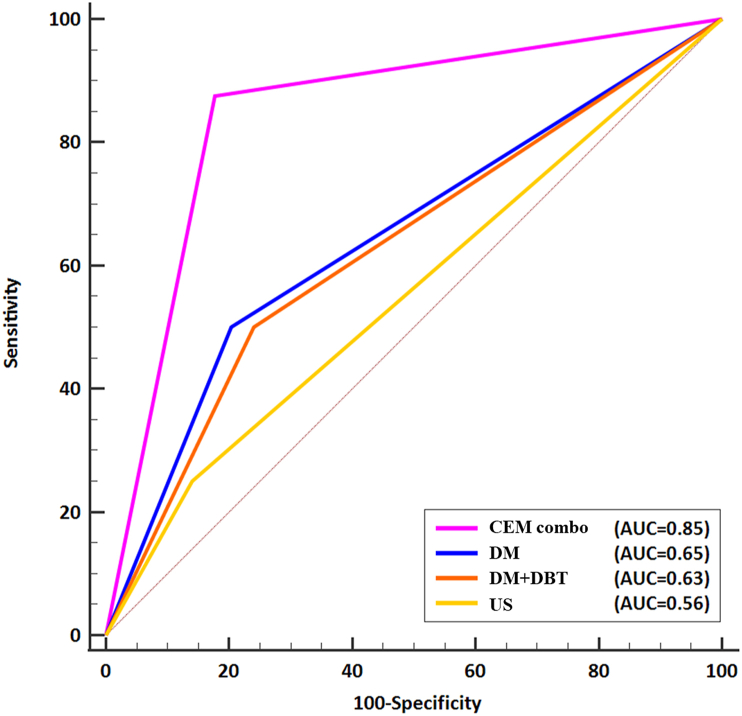
Receiver operating characteristic (ROC) curves comparing different imaging techniques for detecting breast cancer during screening, including DM, DBT, CEM combo, as well as US for their efficacy in detecting breast cancer during screening. The area under the curve (AUC) of the ROC curves for CEM combo was significantly higher than those for DM, DBT, and US, with p-values of 0.029, 0.017, and 0.002, respectively.

Among the diagnostic imaging tools for the diagnosis of breast cancer in 308 screening women, the CEM combo demonstrated the highest sensitivity at 87.5 %. Additionally, CEM had the highest PPV at 11.9 % and the highest NPV at 99.6 % among all modalities evaluated in the study (Table 3). DM demonstrated a sensitivity of 50.0 % and an accuracy of 78.9 %, with a PPV of 6.2 % and a NPV of 98.4 %. DBT had a sensitivity of 50.0 %, identical to DM, and an accuracy of 75.3 %. US had the highest specificity of 86.0 %, but the lowest sensitivity of 25.0 %, the lowest PPV of 4.6 %, and the highest accuracy of 84.4 %. Fig. 3 shows the ROC curves for each imaging modality, and Table 3 lists the respective AUCs, which signify diagnostic effectiveness. ROC curve analysis showed that the CEM combo had better diagnostic performance than DBT, DM, and US. CEM combo had a statistically significant AUC improvement of 0.20 (p = 0.029) over DM, 0.22 (p = 0.017) over DBT, and 0.30 (p = 0.002) over US.

**Table 3:** Diagnostic performance of different diagnostic imaging tools for the diagnosis of 8 women breast cancer in 308 screening women.

Parameter	CEM combo (95 % CI)	DM (95 % CI)	DBT (95 % CI)	US (95 % CI)
Sensitivity (%)	87.5 (47.4, 99.7)	50.0 (15.7, 84.3)	50.0 (15.7, 84.3)	25.0 (3.19, 65.1)
Specificity (%)	82.7 (77.9, 86.8)	79.7 (74.7, 84.1)	76.0 (70.8, 80.7)	86.0 (81.6, 89.7)
PPV (%)	11.9 (4.9, 22.9)	6.2 (1.7, 15.0)	5.3 (1.5, 12.9)	4.6 (0.6, 15.5)
NPV (%)	99.6 (97.8, 99.9)	98.4 (95.8, 99.5)	98.3 (95.6, 99.5)	97.9 (95.1, 99.2)
Accuracy (%)	82.8 (77.1, 86.8)	78.9 (73.9, 83.3)	75.3 (70.1, 80.0)	84.4 (79.9, 88.3)
AUC	0.85 (0.80, 0.89)	0.65 (0.59, 0.70)	0.63 (0.57, 0.68)	0.55 (0.49, 0.61)

Note: Data in parentheses are the 95 % confidence intervals (CI). CEM=Contrast-enhanced mammography, DM = Digital mammography, DBT = Digital breast tomosynthesis, US =Ultrasound, PPV = Positive predictive value, NPV =Negative predictive value, and AUC =Area under curve.

## 4 Discussion

In this prospective study, 308 women with a higher risk of breast cancer underwent CEM screening. CEM exams identified suspicious lesions (BI-RADS 4 or 5) in 19.5 % of women, successfully detecting breast cancer in 7 out of the 8 diagnosed cases, corresponding to an 87.5 % detection rate. CEM had a specificity of 82.7 %, and an accuracy of 82.8 %. In contrast, DM and DBT each identified only 50 % of the cases, while US had a detection rate of just 25 %. The study demonstrated that CEM is a promising tool for breast cancer screening among women with a higher risk, as it has a higher sensitivity than non-enhanced mammographic imaging and breast US.

Although personal and family risk factors were considered for case enrollments, the study further assessed the lifetime risk of the enrolled women using the Gail model, a well-established and validated risk assessment instrument. This model was employed to categorize participants based on their likelihood of developing breast cancer. Notably, the average Gail score in this study was 13.4 %, which falls below the usual high-risk threshold of 20 %. Despite this, the score still indicates a higher lifetime risk compared to the general population, suggesting that this cohort is representative of the typical demographic observed in common clinical settings.

DM has lower sensitivity (25–59 %) in detecting breast cancer in women at higher risk, especially those with dense breast tissue [14]. DBT can identify more breast cancer presenting as architectural distortion and focal asymmetry lesions [15], but it is not as sensitive as CEM for detecting suspected breast cancer lesions [16]. Several studies have found that CEM has a higher sensitivity, specificity, and accuracy for the diagnosis of breast cancer in women with suspected breast cancer lesions than DM [14,16]. CEM has been associated with an increased number of women recommended for biopsy and BI-RADS 3 short-term follow-up compared to DM [10]. This would decrease the diagnostic specificity and PPV of biopsies when CEM is used for screening [10]. However, considering the increased number of early-stage cancers detected, an increase in the number of biopsies may be a necessary trade-off. In our study, CEM increased breast cancer detection by 37.5 % and detected cancers missed by DM alone. This is comparable to the screening results between DM and breast MRI in other studies [14,17]. Therefore, CEM must weigh the benefits of increased cancer detection against the risks of false-positive results.

CEM screening has also been applied to patients with a history of high-risk lesions from prior biopsies [18]. Previous research by Hogan et al. found that CEM had a high sensitivity of 100 % and specificity of 88 % for breast cancer screening in patients with a history of lobular neoplasia [18]. Among women with risk factors for breast cancer such as dense breast tissue and a personal or family history of the disease, the detection rate for CEM was 31.1 per 1000 women and 18 per 1000 women for DM [19].

Among women at high risk with extremely dense breasts, DBT has shown lower rates of advanced cancer compared to DM, with rates of 0.27 versus 0.80 per 1000 exams [20]. We found that the cancer detection rate per 1000 women was 12.9 for DM and DBT, compared to 22.7 for CEM, which is above the average rate for women at average risk. Additionally, six women underwent breast MRI to further evaluate findings only seen on CEM. Among these six women, three were reclassified as BI-RADS 3, and shorter-term follow-up is suggested instead of biopsy. Breast physicians should be aware that second-look or additional US or MRI tests may lead to an increased or decreased number of benign lesion biopsies being needed when considering CEM screening for high-risk women [21,22].

The rate of BI-RADS 3 findings in CEM was 14.9 % in this study, which is higher than the rate of 2.8 % reported in a previous study [10]. However, this is similar to the rate reported in a study of high-risk women undergoing breast MRI, which varies between 7 % and 25 % [23]. The higher rate of BI-RADS 3 findings in CEM may be due to the presence of small, enhancing lesions with imaging features that are not specific for cancer. Additionally, the lack of recommended imaging features for BI-RADS 2 lesions in CEM could lead to increased uncertainty for radiologists. However, the specificity of CEM for women without breast cancer was 82.3 %, which is higher than DM (79.7 %) and DBT (76.0 %). This suggests that CEM is an effective tool for detecting breast cancer, but it is important to be aware of the potential for false positives. Highly sensitive screening methods like CEM risk overdiagnosis, leading to unnecessary biopsies, patient anxiety, and increased costs. Future research should refine diagnostic criteria and use risk stratification to balance accuracy and clinical utility. In our study, DBT was more likely to detect benign architectural distortion lesions and circumscribed masses than DM. However, further studies are needed to determine the most effective combination of DBT and CEM.

CEM has been used in our hospital since 2012 to confirm abnormalities found in women who have had abnormal screening DM or DBT exams [16]. In Taiwan, there is no clear recommendation for screening higher-risk women for breast cancer. However, the American College of Radiology (ACR) recommends annual MRI screening for women with a personal history of breast cancer, dense breasts, or breast cancer diagnosed before the age of 50 [24]. For those unable to access MRI due to cost constraints or contraindications, CEM has the potential to be an alternative method based on increased blood flow of breast malignancies [16].

While CEM demonstrates superior diagnostic accuracy over conventional mammography, its broader application is limited by several factors [25]. The technique requires increased radiation exposure and the use of an iodinated contrast agent, which introduces potential safety concerns for patients. Additionally, the relatively high costs, need for specialized equipment, and requirement for specifically trained personnel restrict its widespread use. Breast cancer mortality is influenced by age, tumor size, lymph node involvement, metastasis, cancer stage, relapse, and hormone receptor status [26]. More extensive research is needed to determine CEM's effectiveness across varied breast densities and to understand its long-term effects on breast cancer mortality rates. Meanwhile, it is essential to explore the risks associated with overdiagnosis and the possibility of undetected malignancies [10,19].

The study had several limitations. First, it was prospectively conducted at a single institution, so the results may not be generalizable to women from other settings. The study population was mostly women with dense breasts and an increased risk of breast cancer due to family history. The performance of CEM may vary slightly among women with different breast cancer risks. Lastly, the study was relatively small, with only 308 initial CEM screenings. Despite its limitations, this research serves as a foundational study for CEM breast cancer screening in Taiwan, highlighting the need for future, multi-institutional research to confirm these preliminary findings and develop standardized screening protocols. Additionally, integrating machine learning algorithms could enhance accuracy, streamline analysis, and improve predictive insights for breast cancer screening.

## 5 Conclusion

CEM effectively detects breast cancer in higher-risk Taiwanese women. Additional research is needed to determine its impact on false-positive rates and biopsy recommendations.

## CRediT authorship contribution statement

**Chen-Pin Chou:** Writing – review & editing, Writing – original draft, Validation, Supervision, Project administration, Methodology, Investigation, Formal analysis, Data curation, Conceptualization. **Yu-Ting Hong:** Writing – review & editing, Writing – original draft, Validation, Supervision, Project administration, Methodology, Investigation, Formal analysis, Data curation, Conceptualization. **Yun Lin:** Validation, Investigation, Formal analysis, Data curation. **Pei-Ying Lin:** Validation, Investigation, Formal analysis, Data curation.

## Institutional Review Board statement

The study was conducted in accordance with the Declaration of Helsinki, and approved by the Institutional Review Board of the Kaohsiung Veterans General Hospital (IRB: KSVGH18-CT11-17, Clinical Trials: NCT05797129).

## Data availability statement

The datasets generated or analyzed during the study are not publicly available due to their containing information that could compromise the privacy of research participants but are available from the corresponding author on reasonable request.

## Ethical statement

The study was conducted in accordance with the Declaration of Helsinki, and approved by the Institutional Review Board of the Kaohsiung Veterans General Hospital (IRB: KSVGH18-CT11-17, Clinical Trials: NCT05797129).

## Funding

This work was supported by grants from 10.13039/501100011913Kaohsiung Veterans General Hospital Research Found (10.13039/501100011913KSVGH 108–158). The study design, data collection and analysis, decision to publish, and preparation of the manuscript were conducted independently and without any involvement from the funders.

## Declaration of competing interest

The authors declare that they have no known competing financial interests or personal relationships that could have appeared to influence the work reported in this paper.
