# Supplementary material for: Screening of breast cancer in higher-risk Taiwanese women using contrast-enhanced mammography
Source: Heliyon. 2025 Jan 10;11(2):e41851. doi: 10.1016/j.heliyon.2025.e41851 (PMC11782981; doi:10.1016/j.heliyon.2025.e41851)
Supplement: Multimedia component 1 [file mmc1.docx]

Mammography Screening Questionnaire

# Personal Information (To be filled out by the woman undergoing the screening)

Name:

National ID Number:

Foreign ID Number (for foreigners):

Date of Birth: Year Month Day

# Clinical Information (To be filled out by the woman undergoing the screening)

## Medical History:

Have you had any of the following diseases?

□ Yes, specify disease:

- □ Benign breast disease

- □ Breast cancer

- □ Other cancer:

□ No

## Family History:

Do any of your blood relatives have breast cancer?

□ Yes, please fill in the table below:

- Relationship: Mother, Sister, Daughter, Grandmother, Maternal Grandmother

- Number of relatives with breast cancer:

□ No

## Menstrual History:

Age at first menstruation: years

Have you gone through menopause?

□ Yes, age at menopause: years

- Reason for menopause:

- □ Natural menopause

- □ Uterus removal

- □ Ovary removal

- □ Other:

□ No

## Reproductive History:

Number of births:

Have you breastfed for at least one month with any child?

□ Yes

□ No

Age at first full-term birth:

## Medication History:

Have you taken hormone replacement therapy?

□ Yes, started at age: , for years

□ No

Have you taken oral contraceptives?

□ Yes, started at age: , for ___years

□ No

## Do you feel any abnormal lumps?

□ Yes, if you feel a lump or pain, please mark "X" on the corresponding position in the diagram:

□ No symptoms

□ Never performed breast self-examination

## Other:

Have you had any of the following examinations in the past two years? (Check all that apply)

□ Clinical breast examination

□ Mammography X-ray

□ Breast ultrasound

□ None of the above

Have you ever had breast surgery?

□ Yes (□ Left side, □ Right side)

□ No

## Allergy to contrast material:

□ Yes (When: , Brands of contrast material: )

□ No

## Renal function:

□ Normal

□ Impaired (need to check renal function before test)
